# ‘Violence exists to show manhood’: Nepali men’s views on domestic violence – a qualitative study

**DOI:** 10.1080/16549716.2020.1788260

**Published:** 2020-07-20

**Authors:** Kunta Devi Pun, Tine R. Tjomsland, Jennifer J. Infanti, Elisabeth Darj

**Affiliations:** a Kathmandu University School of Medical Sciences, Kathmandu University, Dhulikhel, Nepal; b Department of Public Health and Nursing, NTNU: Norwegian University of Science and Technology, Trondheim, Norway; c Department of Obstetrics and Gynecology, St. Olav’s Hospital, Trondheim, Norway; d Department of Women’s and Children’s Health, Uppsala University, Uppsala, Sweden

**Keywords:** Domestic violence, masculinity, focus group discussions, men’s perceptions, Nepal

## Abstract

**Background:**

There is significant evidence of the prevalence and factors associated with domestic violence in high and low-income country settings. However, men’s views on domestic violence are still understudied and have never been reported in Nepali society.

**Objective:**

The aim of the study was to explore Nepali men’s perceptions regarding domestic violence in their communities.

**Method:**

The authors undertook a qualitative study using focus group discussions.

**Results:**

Sixty-six married men, considered they were responsible for upholding family discipline and physically demonstrating their masculinity, and they suggested that violence was a mean for achieving this. Men’s frustration when unable to fulfil their family’s expectations or material needs, and cultural attitudes that precluded interference in other families’ lives, were perceived as factors contributing to domestic violence. The men held the opinion that women also perpetrated domestic violence. Some men were reluctant to accept domestic violence as a norm in Nepali families and were aware of recent changes in societal expectations regarding gender roles.

**Conclusion:**

Overall, the Nepali men who participated in the study held different and sometimes opposing views on domestic violence, ranging from violence justified as a necessity for family discipline, uneasy acceptance that violence was commonplace in families, to definite opposition to the use of domestic violence. The study’s findings provide information for identifying points of intervention for violence-prevention efforts and strategies to alter the social and cultural norms that lead to acceptance of domestic violence.

## Background

Violence against women and girls is a prevalent violation of human rights. It is estimated that one-third of all women globally experience violence at some time in their lives, and the highest prevalence of such violence is in South Asia and on the African continent [1]. Violence against women is often rooted in gender norms and attitudes that subordinate women in societies and simultaneously it serves to maintain an unequal balance of power between men and women. Violence that directly targets women takes many forms in public and private life, and is likely to result in physical, sexual, psychological, or economic harm or suffering, including threats of such acts and coercion or arbitrary deprivation of liberty [2]. In South Asia, one of the most common forms of violence is domestic, which refers to violence used by women’s intimate partners or other family members [3]. Worldwide, the consequences of domestic violence can be as severe as suicide and homicide [4,5]. In this article, we focus on domestic violence in Nepal, which is highly prevalent, including in pregnancy [6,7].

The risk factors for experiencing and perpetrating domestic violence are complex. Social and cultural norms are among the most important contextual factors. Norms also influence the effects or consequences of domestic violence, the characteristics of the violence, and prevention and mitigation efforts. Social and cultural norms are informal rules that prescribe which behaviours are expected, allowed, or sanctioned in particular circumstances [8–10]. Upon marriage, most Nepali women move into their husband’s house in joint or extended families and are expected to assume responsibility for caring for the household and the needs of their parents-in-law. The women typically have domestic and agricultural responsibilities and are financially dependent on their husband’s income [7]. As a consequence, the kinship system in Nepal can leave women vulnerable to controlling behaviours by their husband or other family members [11]. Previous studies have identified the existence and influence of socially sanctioned behaviours in Nepal that are linked to women’s risk of experiencing domestic violence, and include the following: men’s ownership of their wives through dowries or bride wealth; a social system that attributes greater value to sons than daughters; expectations that men provide financially for their families while women maintain domestic harmony; family shame associated with divorce; and lack of faith in the justice system, which inhibits formal reporting of domestic violence [7,10].

Most efforts to address and mitigate the factors in domestic violence have focused on women [12,13]. However, men are increasingly engaged in preventing and reducing violence against women on a global scale [14]. In a few cases, researchers have studied men in their research on the social norms, attitudes and behaviours that allow for the continued high prevalence rates of domestic violence in South Asia. For example, in a study conducted in Sri Lanka, the majority of 1568 male participants agreed with the statement ‘There are times when a woman deserves to be beaten’ [15]. The men associated manhood with dominance and agreed that ‘It is manly to defend the honour of your family, even by violent means.’ One-quarter of the participants were of the opinion that women should accept teasing of a sexual nature because ‘it is harmless’ [15]. A qualitative study from Bangladesh found that men were heavily influenced by patriarchal norms and regarded their wives as their property. They were of the opinion that a wife should obey her husband, and that it was acceptable to beat a wife for failing to fulfil their (the husbands’) expectations [16]. Globally, men are the most common perpetrators of domestic violence and therefore it is important to increase knowledge of their perceptions of the various factors that underlie their use of violence against women in different contexts. Such knowledge can be used to identify points of intervention for violence prevention and culturally appropriate strategies to alter norms that lead to the acceptance of domestic violence, as well as bolster existing social norms that protect women. Accordingly, the purpose of this article was to explore Nepali men’s perceptions regarding domestic violence in their communities.

### Theoretical framework

There are various definitions and perspectives on the attributes, interests, and attitudes that men are expected to display in social situations, including in their families. For example, hegemonic masculinity is a persistent and socially constructed view that legitimizes men’s dominant positions in families and societies, and defines men by attributes such as physical strength, emotional control, and providing materially for family needs [17]. The roles expected of men vary across cultures and between individuals. Previous studies have shown that in the social environments of our study context, hegemonic masculinity is pervasive [6,7]. We reflected on our findings during the analysis using a socio-ecological theory, where the results could illustrate the complexity in this context between individual views, perceptions of relationships, family expectations and the community’s potential to reduce domestic violence [18,19].

## Method

### Study design and setting

We chose a qualitative design and used focus group discussions (FGDs) to achieve the aim of improving the understanding of men’s perceptions regarding domestic violence in Nepal. A strength of using FGDs is that the participants have the possibility to collaboratively explore issues that are not well understood or are also understudied [20]. We did not aim to obtain individual histories of the experiences or use of violence. The study was carried out over the course of four months in 2014, in community centres in seven different wards within Dhulikhel Municipality and located 30 km east of Kathmandu. In total, the municipality comprised 3279 households and 14,283 people. Dhulikhel Municipality is primarily a semi-urban area, with one larger centre with a hospital and schools [21]. Previous studies have investigated pregnant women’s perceptions and prevalence of domestic violence in Dhulikhel and we wanted to add men’s perceptions to understand their views on this phenomenon [6,7]. Most of the inhabitants in the villages are engaged in agriculture. There are several ethnic groups and c.80 languages are spoken in Nepal but in Dhulikhel most of the inhabitants are from the Newar ethnic group and speak Nepali. The men in the area are more literate (88.6%) than the women (70.3%) [21].

### Study participants

The first author, with support from social mobilisers, invited Nepali men to voluntarily participate in FGDs regarding domestic violence. The men were informed of the study during their regular biweekly community meetings. We purposefully invited men with different ages, occupations and levels of education; illiterate, primary school, secondary school, SLC (School Leaving Certificate, obtained after finishing 12 years of education) or university. The inclusion criteria were married men, 18 years of age or older, and agreeing to participate (Table 1). Only a few men declined the invitation to attend, mainly from one of the FGDs, with 5 participants, as they were too busy or otherwise lacking time. All participants were married, 11 were grandfathers, and all had children, except for 8 young men. Ninety-two (56%) of the children were sons and, 71 (44%) daughters. Most of the participants were either farmers (36%) or worked in the service or business sectors (33%). Of 66 participants, 18 were illiterate or could read and write simple sentences.

**Table 1.** Profile of participating Nepali men.

Group	Age	Education	Occupation	Number of participants
1	27–52	illiterate; primary and secondary school, SLC	farmers	10
2	22–44	can read & write; primary and secondary school	farmers; service; business	10
3	35–81	Illiterate, can read & write; primary and secondary school; SLC	farmers; service; business	10
4	18–40	can read & write; secondary school	service; business; unemployed	7
5	32–47	university	teachers; clinicians	5
6	26–58	primary and secondary school; SLC	farmers; service; social work; job abroad	8
7	34–65	can read & write; primary school; SLC	service; business	7
8	34–65	can read & write; secondary school; SLC; university	business; service; teachers	9

Can read and write; able to understand simple sentences, SLC; School Leaving Certificate (finished 12 years of education).

### Data collection processes

The first author moderated all FGDs and an observer took notes. A total of eight FGDs were held, with 66 men, in convenient private areas where the men usually meet. There were 5–10 men in each group. The men were asked if they had any grandchildren. Men reporting at least one grandchild were allocated to groups called ‘older men’. The younger men, those without grandchildren, participated in separate group discussions from the older men to avoid potential age-related domination in social relations among the participants. However, the youngest grandfather was 34 years old, and the oldest man without a grandchild was 52 years old, which meant the age ranges were wide and overlapping within both groups. Each FGD lasted an average of 50 minutes (34–60 minutes). The discussions, conducted in Nepali, were audio-recorded with the participants’ consent. An interview guide was used, covering topics regarding men’s perceptions of gender roles in family relationships, health consequences of domestic violence, violence during pregnancy, and possible solutions to ending violence against women. The discussion started with an open question for the men to discuss: *We live in a community, both men and women. What are your views on gender roles in family relationships in Nepal?* After six FGDs, we perceived that few new insights were being gained, but we carried out a further two FGDs before judging that we had reached data saturation. The audio-recordings were transcribed and translated into English, and some FGDs were back-translated to check for accuracy. The FGDs were coded by numbers (e.g. FGD1, FGD2) and the individual participants were identified by their own stated education and age.

### Data analysis

We followed the processes of content analysis described by Graneheim and Lundman [22] to organise and elicit meaning from our data. The approach allowed us to reduce the volume of text and to identify groups of related meanings. Initially, the first author’s interpretations of the overarching insights and meaning in the data were checked with the observer to identify any possible misunderstandings. While interpreting the data, the first author reflected continually on her positions as a Nepali daughter, wife, daughter-in-law, mother and midwife.

We carried out an inductive content analysis at a descriptive manifest level [22]. This entailed the authors reading all the transcripts of the FGDs to identify content areas, and find meaning units from the text, which were reduced and condensed. By abstraction the condensed units were coded, using NVivo 11. The latent content of the codes were grouped together and labelled as categories. This process continued until we reached agreement on descriptive themes, which illustrated common threads in the FGDs (Table 2).

**Table 2.** Examples of the analytic process derived from Focus group discussions.

Content area	Meaning unit	Condensed meaning units	Codes	Categories	Descriptive theme
Traditional family norms that influence the occurrence of violence	Everyone blames men, but it is not like that Men are also violated This does not come out, because our country is male dominated, so a man hesitates to express that he is violated by a woman	Blaming men Men are violated A man hesitate to express he is violated	A man should discipline his family Both genders are violent Expected male dominance	Families’ expectations Cultural acceptance that both men and women are violent	Questioning putting the sole responsibility for violence on men

## Results

Four different descriptive themes were developed based on ten categories. They are here presented under their respective content areas (Table 3). **Social norms and cultural practices that are harmful to women**

**Table 3.** Nepali men’s perception of social and cultural norms related to domestic violence.

Content areas	Categories	Descriptive themes
Social norms and cultural practices that are harmful to women	Son preference Religion, cultural practices and gender inequity The meaning of being a man	Considering gender socialization and norms which threaten women’s safety
Traditional norms within families that influence the occurrence of violence	Families’ expectations Cultural acceptance that both men and women are violent	Questioning putting the sole responsibility for violence on men
Social norms that protect women from domestic violence	Pregnancy – a period of protection? Changing social norms Women are gaining independence	Seeing a transition of gender norms and roles
Continuing challenges that may undermine the positive trends in changing social norms	Uncertainty about the right to intervene when witnessing other men’s violence Reduction in domestic violence is needed but it is also important to preserve Nepali traditions	Acknowledging the conflict between need for change and maintaining Nepali traditions

### Considering gender socialisation and norms which threaten women’s safety

Social norms, such as son preference, the prominence of men in religious ceremonies, and patriarchal expressions of masculinity may translate into harmful practices and can be interpreted as culture-specific domestic violence.

#### Son preference

The greater value attributed to having a male child rather than a female child was recognised in all FGDs, and one traditional practice is to serve better food after a boy is born compared with when a girl is born:

Yes, this boy and girl issue is a bizarre thing. Even educated families wish for a son … [The importance of] giving birth to a son is deeply rooted. (FGD 5, university, 36 years)

That is due to our culture and tradition which we cannot leave. (FGD 5, university, 47 years)

There is a saying as well, that if a son is born, we celebrate it with goat’s meat. Otherwise, we eat pumpkin for a girl. There is a different way of looking at a daughter, still. (FGD 7, can read & write, 35 years)

Additionally, men were aware of sex-selective abortions of girl foetuses, and prospective parents were advised to try for a son instead later.

#### Religion, cultural practices and gender inequity

When the study participants discussed whether the patriarchal use of violence in Nepal stemmed from religion or culture, the strong son preference was attributed to religious beliefs, as sons are required for ancestors to transcend to the next world:

We have a belief that if we have no son, then we or our ancestors can’t transcend to another world after death. (FGD 7, can read & write, 35 years)

This is due to what is written in Hindu scriptures … [but] also found in Buddhism … Religion has given men privileges … for example, the Goddess of Beauty and Wealth, Laxmi [Lakshmi], sits at the foot of Lord Bishnu [Vishnu]. The impact still exists even these days. (FGD 6, secondary school, 43 years)

In Nepal, a son is expected to bring to his parents’ happiness and security, to look after the elderly, to support the family, and to bring a daughter-in-law into the house. After marriage, it is expected that a son should be born. The study participants identified the lack of a son or infertility as justifications for domestic violence:

They may experience physical violence for giving birth to a daughter. Moreover, if she has already one or two daughters, we cannot imagine how much she is tortured for having another [daughter]. (FGD 5, university, 47 years)

Although government laws prohibit marriage before 18 years of age, early marriages are still common and young women may not have sufficient life experiences or capability to manage a household, which is expected by her new family. The participants in the FGDs discussed how such young girls are particularly scolded and treated violently:

We have the culture of marriage at a young age. This way of marrying is also a [type of] violence. (FGD 2, secondary school, 27 years)

#### The meaning of being a man

All participants described men’s frustrations about not being able to fulfil societal expectations as ‘breadwinners’ and support their family in economic terms, which often transformed into quarrels that escalated into violence in their home. A common perception expressed in the discussions was that domestic violence was caused by family discontent with traditional expectations of men, as well as men’s attempts to express their masculinity:

Violence exists to show manhood. As a man, I should make women work a lot … there is a strong feeling that the women’s work and the men’s work are separate. There is no equality [in tasks] … It is not like beating too hard till she gets hurt or wounded (Ha! Ha!) … We [only] beat when it gets too much. (FGD 1, secondary school, 38 years)

Further examples of family dissatisfaction and frustration linked to gender roles and expectations were identified as stemming from poverty, lack of education, widespread unemployment, illiteracy, and alcohol abuse:

We drink until we get drunk. In drunken states, quarrels take place and arguments occur. Now, here, most are poor, and in poverty one is unable to fulfil needs. This inability to fulfil needs leads to arguments.’ (FGD 4, can read & write, 26 years)

Lack of community awareness and knowledge about domestic violence, as well as ignorance about the harm that may be caused by violence were frequently brought up in the discussions. The men considered themselves the most important partner in a marriage and they expected the women in their families to do what they said and wanted. In such a patriarchal context, women are inclined to obey and serve their husbands:

If she does not prepare meals at the time asked by the husband, this may lead to violence. (FGD 4, secondary school, 40 years)

If a woman argues, then naturally she should be beaten. (FGD 1, primary school, 23 years)

Yes, [violence is necessary] to discipline. To be truthful, we should agree, that beating is necessary. First, we try to make her understand. We are humans after all, we get angry … We beat to discipline her. (FGD 1, secondary school, 38 years)

## Traditional norms within families that influence the occurrence of violence

### Questioning putting the sole responsibility for violence on men

Traditional societal norms spill over into familial norms and expectations of a new young wife in her husband’s family were discussed at length in the FGDs. However, some men reacted negatively to the common belief that they were the only perpetrators of violence in families.

#### Families’ expectations

In joint and extended families, in-laws play an important role in the well-being of a new wife and in-law’s expectations can often leave women in a vulnerable situation. Some participants held the opinion that in-laws often arranged their son’s marriage primarily to ensure they would have help in the household and they might treat the wife poorly. However, others expressed that this was how the family system functioned in Nepal:

Women need to understand it is their own fate, like a lottery, some land in a better house and some in a house where they have to suffer a little. (FGD 1, primary school, 23 years)

In-laws may lack education and there may be simple misunderstandings that may later become violence. (FGD 2, secondary school, 34 years)

They [in-laws] do it [beating] because they lack knowledge. (FGD 1, secondary school, 45 years)

An increasingly common situation in Nepal is when brothers-in-law and other men in the family take advantage of a new wife in the family while the wife’s husband works abroad. However, a number of participants in the FGDs rejected the notion that in-laws always treated a new wife poorly. They clarified that many in-laws treated the new wife well, as a daughter:

Daughters-in-law are like our daughters, so we must love them. Yes, we must love another’s daughter when she is brought to our house after our son marries her. (FGD 3, illiterate, 35 years)

The material and social circumstances in Nepal that result in women being financially and socially dependent on their husbands may create tension. Some men identified this as a way of ‘making’ women passive or without will or desire to take any initiative, but others suggested that this type of dependency might be beneficial for husbands and hence desired by them:

Women are dependent on others … It is not like they are forced to depend on others. Rather, it is our culture that has taught women to depend on men … and we like that, too. (FGD 5, university, 32 years)

Women without education, particularly those who live in rural areas, may not be able to leave a violent relationship or they may consider wife-beating as a husband’s right. It is still prohibitively difficult for most women in Nepal to divorce:

If a woman’s family is broken, even the maternal family will not look after her again. She will become homeless. [To avoid this], she will rather suppress herself and try to remain without a word. (FGD 5, university, 36 years)

Some participants in the group discussions recognised that this might be an unbearable situation for some women, possibly even leading women to suicidal thoughts or attempts at suicide. Women’s suicide by poisoning, mainly by drinking pesticides in the fields, was well known to the study participants:

If one has to suffer violence regularly, then it is natural to think of dying. (FGD 1, secondary school, 38 years)

#### Cultural acceptance that both men and women are violent

The participants discussed how men were unfairly blamed as the sole perpetrators of domestic violence in Nepali society and that domestic quarrels were often begun by women. Mothers-in-laws were identified as often accusing fathers-in-law of helping their daughters-in-law ‘too much’ or even of being attracted to them and in such situations, they blamed the daughters-in-law. Furthermore, the mothers-in-law asked their sons to resolve the perceived problems by beating their wives, and then fathers-in-laws sometimes had to act to protect their daughters-in-law against the mothers-in-law or sisters-in-law. Some men said that as husbands, they constantly experienced the need to find a balance between satisfying the wishes and expectations of their parents, siblings and wives, and often they ended up beating their wives.

Mothers-in-law were identified as treating their daughters-in-law especially badly when their sons were working abroad to earn money:

Females are the reason for violence against women, which men may not notice. (FGD 2, can read & write, 42 years)

Some participants believed that mothers-in-law ‘managed’ the family, while others perceived that fathers-in-law were the most dominant in-laws, as both controlled their wives and other members of the family. In either situation, when one of the in-laws has the most power in a family, the daughter-in-law has no option but to accept violence if it occurs, as it is perceived that she will be showing respect for members of the older generation, which is most important. Some participants also suggested that women are physically violent towards other women and men:

Everyone blames men, but it’s not like that. Men are also violated. This does not come out, because our country is male dominated, so a man hesitates to express that he is violated by a woman. (FGD 4, can read & write, 44 years)

Yes, yes, a wife beats. (FGD 1, illiterate, 71 years)

In the context of discussions about women’s violence, divorce was again a topic that preoccupied many of the participants in the FGDs. After a divorce, a woman is entitled by law to half of the property from the marriage. The participants discussed rumours that some women repeatedly eloped with different husbands in order to gain land from several men. The men were worried about losing property if they divorced and they described the threat of divorce as a form of violence from women against men and as a reason for men to perpetrate acts of violence against women:

These days, some of the women are marrying three or four men and claiming property from everyone. (FGD 4, can read & write, 44 years)

One of my friends … bought a house, land and car for her. When he returned from abroad … the wife had sold all the property and gone off with some other person, and no one knows where she is … now he doesn’t have a rupee. (FGD 4, secondary school, 27 years)

## Social norms that protect women from domestic violence

### Seeing a transition of gender norms and roles

Women may need to reduce their workloads and need nourishing food for optimal pregnancies. Men perceived that living in an educated family was easier for pregnant women in Nepal. Otherwise, women would be required to continue with their heavy workloads, often in agricultural settings, as well as in their homes during pregnancy.

#### Pregnancy – a period of protection?

In the FGDs, many of the participants clearly expressed that physical violence by husbands should be avoided during pregnancy because it was considered a sin. They were aware of potential consequences of physical violence, such as miscarriage and serious emotional distress caused to women. Men who beat pregnant women were not respected and some of the participants held the opinion that men should help their wives with household chores during pregnancy:

Yes, we make her do a smaller amount of work [during pregnancy] and give her green vegetables to eat. (FGD 7, primary school, 46 years)

The baby could be handicapped or die inside the womb [due to physical violence] … and, the woman, she may die or … suffer mentally and psychologically. (FGD 8, secondary school, 41 years)

However, mothers-in-law were identified as being potentially cruel during the pregnancies of their daughters-in-law. In some discussions, the men explained that it was common practice for mothers-in-law to deny pregnant daughters-in-law the opportunity to attend antenatal care, to restrict the amount of nourishing food they received (if they were not working ‘hard enough’ during pregnancy), and make the daughters-in-law work hard immediately after having given birth.

#### Changing social norms

Patriarchal social norms are currently changing, and some participants held the view that gender inequalities were consequently diminishing. For example, it is now possible for a woman to be a political candidate and elected for a political office, and many cultural expectations of female widows are no longer customary or legal, such as prohibitions on obtaining travel documents, which formerly required a husband’s signature. Previously, widows wore white saris, but today they can also wear other bright colours. Furthermore, parents-in-law may look for a new husband for their daughters-in-law if their son has died, so that she can remarry.

#### Women are gaining independence

The younger generations in Nepal can choose to live separately from their parents, in nuclear families rather than extended families. This often involves a couple working together to complete household chores and to avoid potential mistreatment from in-laws. Furthermore, women are more independent today than women were in previous generations, as they have their own careers outside the home, which leads to a better economy for the entire family and reduces tensions between generations:

Men and women work together [now] … educated husbands and wives are working equally. (FGD 7, primary school, 46 years)

However, new ways of living were mentioned as also negatively affecting the older generations:

There will be no one to care [for the elderly]. (FGD 7, School Leaving Certificate (SLC), 34 years)

Once you educate them, they go abroad, such as to Europe, to earn some money, come home and build a separate house. (FGD 6, SLC, 55 years)

Traditionally, funeral rituals were only performed by sons and men, but this custom, too, is changing:

I know [someone] who decided to give all his properties to his daughters, and they will do the rituals after [his] death. (FGD 7, primary school, 46 years)

Boys and girls are now integrated in the same schools and consequently girls are receiving more education than previously, which has led to positive changes:

In comparison to in the past, boys nowadays are better behaved … they don’t tease and be naughty [to girls]. They attend boarding school and become educated … boys and girls are united and treat each other equally in the classes. (FGD 8, secondary school, 65 years)

## Continuing challenges that may undermine the positive trends in changing social norms

### Acknowledging the conflict between need for change and maintaining Nepali traditions

Although societal norms are evolving, there are still challenges with respect to addressing domestic violence.

#### Uncertainty about the right to intervene when witnessing other men’s violence

Some of the study participants explained the difficulty of not feeling entitled to help or interfere to reduce the amount of men’s violence against women. Domestic violence was generally considered a private family matter and interference was unwanted or even risky. Wives were seen as tolerating beatings by their husbands due to their dependency on the family, and a number of the men were of the opinion that women’s situations became even more complex if a third party became involved:

They [the women] turn against us if we try to counsel them. We, ourselves, do not feel safe to intervene. (FGD 7, primary school, 46 years)

I helped her … and her husband slapped me very hard … blood spurted out of my mouth. What could I do? (FGD 7, can read & write, 45 years)

The men discussed how wives sometimes asked the police to release their husbands if anyone had interfered in cases of domestic violence. They understood this as strategic for the women, due to their fear of further violence against them. The participants discussed the conflict they experienced of not wanting to interfere in other people’s family matters, yet not tolerating domestic violence.

#### Reduction in domestic violence is needed but it is also important to preserve Nepali traditions

The men discussed the importance of education to reduce ignorance and increase awareness of consequences of violence. They were of the opinion that if communities and the government increasingly recognised domestic violence, it would give individuals a stronger mandate and motivation to intervene in ‘family matters. Several laws in Nepal are designed to prevent domestic violence or lead to the prosecution of the perpetrators of domestic violence, but a number of the men considered that the laws were either not implemented or not followed, which thus enhanced the continuation of violence. They suggested that future efforts to reduce domestic violence should be made at different levels and must involve men:

We can organise some interaction programmes … mothers-in-law and fathers-in-law can be brought together to raise awareness. (FGD 2, secondary school, 27 years)

We must hold awareness programmes. Make the public understand. (FGD 1, secondary school, 38 years)

Society should cooperate with us men, to bring positive changes. (FGD 7, can read & write, 37 years)

The participants discussed Nepali cultural and traditional influences, and the importance of preserving positive and respected traditions, as well as limiting unwanted influences from the Western world. The men recognised that the country is in a transition period, and some customs are clashing with global world influences:

In our community, slapping our children is not a big deal, but the same activity is considered a big crime in Western culture. (FGD 5, university, 36 years)

There was significant discussion about the complexity of wanting to adopt some Western customs while preserving Nepalese traditions, particularly in relation to the dowry system, whereby wealth from a woman’s family is transferred to the husband’s family upon marriage. The amount of dowry was vividly discussed. If a small dowry were received, the husband might choose not to register the marriage, in which case the woman would not be able to obtain a citizenship card, which would be a violation of her rights. Women with small dowries are treated worse than those with wealthier dowries:

Those without a dowry will be treated like slaves. (FGD 3, can read & write, 45 years)

You may see physical violence caused by the dowry system. (FGD 5, university, 36 years)

## Discussion

An overarching theme in our data is that *Nepali men adhere to traditional gender social norms yet also realise that times are changing*. Connell’s theory of hegemonic masculinity has greatly influenced the concept and interpretation of being a man [17]. Connell saw gender as a system of social inequality, and the social construction of masculinity, often inherited trans-generationally, as linked to the social power of patriarchy. Male domination, however, does not inevitably include all men, and it is a practice that is constantly re-constituted [23]. Our interpretation is that patriarchal hierarchy and normative masculine and feminine roles are strongly prevalent in Nepal. However, we also found that social norms are changing, and some men did not support hegemonic masculinity. During the discussion, we reflected on our findings using a socio-ecological approach, as described by Heise who built on Bronfenbrenner’s theories, that human development is influenced by our social environments [18,19].

From a social-ecological perspective, *individual views* from the Nepali men in the study provide examples of deep-rooted, male-controlled customs that enable and lead to the acceptance of domestic violence. Some of the men acknowledged that they needed to demonstrate manhood within marriage though physical discipline in order to ‘correct’ their wives’ behaviours and to adhere to static social roles. Our findings support the dominant male model, in line with a study conducted in Indonesia and India which, based on 86 interviews, found that men simultaneously denied domestic violence, stating that it rarely happened, yet contradictorily arguing that domestic violence was ‘only natural’ and women deserved punishment for not obeying their husbands [24]. In our study, the men expressed a strong belief that domestic violence targeted at women was related to family frustrations regarding both gender-related and age-related roles and expectations, as well as the family’s material circumstances.

From the FGDs, we interpret that Nepali men who maintained a traditionalist position toward gender and sex roles had the highest level of acceptance of violence and considered violence a means to uphold the superior position of men within marriage. Notable alternative perceptions were also discussed. Men with a more pragmatic view considered violence undesirable but sometimes necessary in order to correct a wife’s behaviour, and men with an egalitarian perspective did not see any reason for violence, because men and women are equal and complementary. In a Javanese study, three different positions of masculinity were identified, all of which are similar to the perspectives held by men in our study [25]. Although we included younger and older men in separate discussion groups in our study, we cannot conclude whether age related to differences in their views. The differences in our study seemed linked more to whether the men were educated.

At a *relational or family level*, from a global perspective, men are more likely than women to be perpetrators of domestic violence [1]. In our study, the men found it challenging to accept all responsibility for domestic violence. They often blamed women for behaviours that provoked violence or blamed in-laws for initiating violence against their wives. They also drew attention to their own exposure to violence from females in their families. It is not uncommon for men to place the burden of responsibility for domestic violence on women [24,26]. This has been recognised as a resistance strategy, particularly in patriarchal contexts, which can result in normalising the violence and diverting attention away from men’s culpability and towards wider structural or cultural factors [26].

The impact of the attitudes of in-laws seems essential for the future of young women who move in with their husbands’ families upon marriage. Respect for and obedience to elders is pronounced in Nepal, as in other societies in which social and gender roles are relatively fixed, and this can have wide-reaching effects on the mental and physical health of the new wives. It is important to consider the impact of these factors on men’s experiences and health too. Attitudes that include acceptance of domestic violence contribute to the transgenerational cycle of violence, and childhood experiences of violence or of witnessing violence in the family are known risk factors for later perpetrations of domestic violence [27]. We observed that the men in our study maintained traditional gender norms but simultaneously acknowledged structural changes in family organisation. The men discussed increasing numbers of nuclear families rather than extended families, as facilitating the abilities of the younger generations to contest traditional gender expectations. In future studies, it would be prudent to evaluate whether and how these kinship transitions relate to reductions in the prevalence of domestic violence.

Our interpretation is that the Nepali tradition of preferring sons over daughters is still highly prevalent. It was discussed in all FDGs, despite the study participants’ perspective that these preferences are evolving. The Nepali men knew of sex-selective abortions, and the birth of daughters was recognised as a cause of violence against women perpetrated by their husbands and/or in-laws. Similar descriptions were provided by community members in our previous study conducted in Nepal [7]. Son preference is a guiding characteristic in many patrilineal societies but is not always associated with abuse of women. Future studies in Nepal could explore the specific contextual factors in Nepal that seem to allow for this type of violence.

We understand that domestic violence during pregnancy is not seen as commonly practised. Pregnancy appears to afford a period of protection from physical violence, although in-laws can have a substantial impact on their daughters-in-laws’ well-being during pregnancy. This was similarly found in a study conducted in Tanzania, where the mothers-in-laws controlled their daughters-in-laws’ access to antenatal care [28].

Furthermore, the perpetration of domestic violence was considered a private matter and external interference to prevent such violence was not welcomed. We may interpret domestic violence as normal family business or a taboo topic, about which discussions should be avoided. In neighbouring China, violence is common, but fear of being socially stigmatised or dishonoured has been found to have emotional and material consequences, and ‘saving face’ was found most important [29].

Culture and religion were discussed on a *societal level*, and their relationship to domestic violence. Many of the participants held the opinion that violence against women should not be accepted and was not an inherent part of Nepali culture or religion. They considered that the influences of globalisation, such as exposure to Western films and access to the Internet were often responsible for domestic violence. Our interpretation is that the complexity of maintaining Nepali traditions and social norms, many of which are patriarchal, while embracing Western influences aimed to reduce domestic violence was evident and a dilemma for many participants. Similar findings have been made in other studies conducted in South Asia. For example, in Sri Lanka, male university students recognised themselves as being at a crossroads between modern and traditional influences [30].

Our Nepali participants suggested common efforts for primary prevention of violence in the community. There are laws in Nepal meant to prevent domestic violence and penalties for perpetrators, but the men in our study perceived that the laws are not followed. They felt that efforts to reduce domestic violence must involve awareness-raising programmes and education in order to increase knowledge of the health consequences of domestic violence. These suggestions are in line with those found in other studies. Two Ugandan interventions programmes have shown promising effects: SASA! and the Good School Toolkit are community and school-based mobilisation programmes that involve families at risk of violence, and both programmes have been effective means of preventing gender-based violence [31,32]. We can interpret from our findings that regardless of existing domestic violence in Nepali society, social norms that support violence, such as cultural acceptance, are changing. The Nepali men in our study held similar perceptions to the men in a study in rural Bangladesh on women’s participation in societal development. The Bangladeshi men feared a loss of male authority, but also recognised positive effects of women’s more independent roles in contributing to the economic welfare of families [33].

### Strengths, trustworthiness and limitations

We have described our choice of methods for data collection as one measure to ensure the *credibility* of this study, which is in turn a component of the study’s trustworthiness. We have stayed closed to our participants own words in our presentation of the study findings in order to capture their realities, and we have incorporated illustrative quotes from the FGDs. Yet we also delve into the underlying meanings, experiences and views of the participants. The diversity of the international research team, representing different countries of origin, life experiences, academic training and professions, also contributes to the study’s *credibility* as it reduces the chances that our individual biases and perspectives have influenced the presentation and interpretation of the findings. Furthermore, it provided us opportunities to compare and challenge our different interpretations of the data; the team has reviewed and verified the study’s evidence. The first author (KDP) is a midwife, fluent in Nepali. KDP, JJI (anthropologist), ED (obstetrician) and TT (medical student) have knowledge of the subject and qualitative research methods, and three of us (KDP, JJI and ED) are familiar with the context. Another strength is that, in this first study among Nepali men, we were able to include participants with varying ages, education levels and occupations. We consider the heterogeneity (Table 1) is a strength as it helps to identify important shared patterns that cut across individual cases. We have described the culture and context of the study setting, the participants, the data collection processes and stages of systematic analysis for readers to be able to assess the *transferability* of our findings to other contexts. *Dependability* is established as our team of researchers is familiar with the Nepali study area, either living there or having spent extended time there, and by regularly adapting the interview guide during the discussions.

A limitation is that the research team was entirely female, and it would have been preferable to involve at least one male Nepali researcher with skills in qualitative methods in the research team. However, none were identified in the area. To address this limitation, we returned to some of the participants in order to discuss our interpretations and to enhance our understanding, but no additional information was obtained.

## Conclusions

The Nepali men who participated in the study held differing views on the existence of domestic violence. Frustration about not being able to fulfil the expectations of men in Nepal was perceived as a significant source of family violence, and external interference in family affairs was neither common nor welcomed. Furthermore, the men attributed responsibility to women for initiating or provoking domestic violence. Female in-laws were identified as wielding power to treat a new wife and/or daughter-in-law badly. However, our participants held the view that the tides of global change, although complex, were leading to some corresponding new customs, such as a move away from extended families to nuclear families, which was reducing the existence of domestic violence in Nepali society. This first study describing Nepali men’s perceptions of domestic violence contributes insights that are relevant for future violence prevention and intervention strategies.

## Paper context

Men’s views on domestic violence have not been studied in Nepal. In focus groups men discussed how cultural and social norms contributed to domestic violence, such as controlling or disciplinary roles assigned to men. They were frustrated when unable to fulfil familial expectations, but they felt women also perpetrated violence. Some discussed changes in societal expectations regarding gender roles. They suggested violence-prevention strategies to alter norms that lead to acceptance of domestic violence.

## Annex 1

**INFORMATION SHEET & TOPIC GUIDE FOR FOCUS GROUP DISCUSSIONS ON MEN’S VIEW ON VIOLENCE AGAINST WOMEN DURING PREGNANCY**

**INTRODUCTION**

Namaste! We are Kunta Devi Pun and _________ (Research Assistant). We are researchers of women’s health from Dhulikhel Hospital-Kathmandu University. At the moment we are here to conduct a study on men’s views about violence against women during pregnancy.

**Purpose of the study**

The purpose of the study is to investigate men’s perceptions on violence against pregnant women in Nepali society. Your opinions are important for us to explore in order to suggest relevant and feasible health care based interventions in the future to improve pregnancy outcomes.

**What participation involves**

This discussion will take approximately 1 to 1.5 hours. We will be discussing about perceptions of violence within society, specifically targeting pregnant women and the consequences of violence during pregnancy. You are asked to discuss freely, there are no right or wrong opinions in the discussion on the subject. You should only share thoughts during the discussion you feel comfortable with the others in the group knowing. In order to be able to remember and interpret our discussion later, a tape recorder will be used, if you agree to this, and written notes will also be taken.

**Confidentiality**

We want to assure you that the discussion will be kept confidential by us and only used for research purposes. We will not keep records of your names and you will not be cited or mentioned using you name or other characteristics that will make you recognizable.

**Risks and benefits**

We do not expect that participating in this discussion will cause you any harm. We will learn by hearing your opinions, and including them in our study, and hopefully this will be for the benefit of others in the future.

**Right to withdraw**

You have been asked to participate in this discussion on the basis that you, as husbands and fathers, play important roles in family life in Nepali communities. Choosing to take part in this study is voluntary. If you choose not to participate or if you decide to stop participating during the discussion, there will be no consequence. You can withdraw at any time, without giving any reason.

**TOPIC 1 – Gender roles in family relationships in Nepal**

We live in a community, both men and women. What are your views on gender roles in family relationships in Nepal?

Probes:

- notions of manhood
- advantages and disadvantages of being a man
- relationships with women/wives
- opinions on men’s domination and women’s subordination in relationships
- family expectations
- roles of mothers-in-law and other family members
- power and decision-making authority in families/other spheres of life for men and women (workplace, schools, social functions, etc.)

**TOPIC 2 – Violence in general**

Sometimes, we may encounter violence in a community. What do men think about violence in general?

Probes - what does it look like in this community?

- meaning
- causes
- types
- is it a serious problem in your community (Dhulikhel)/Nepal?
- whose problem is it? (community, family, individual, etc.)
- who is to blame?
- perpetrators, victims
- who should be responsible for responding to it?

**TOPIC 3 – Violence during pregnancy**

Sometimes, during pregnancy, home may not be a safe place for the pregnant women. What do men think of violence against pregnant women?

Probe for – types of violence experienced by pregnant women

- causes of violence during pregnancy (why it happens)
- cultural norms about how pregnant women are treated – are pregnant women treated differently? Can pregnancy protect women from violence, or cause more violence?

**TOPIC 4 – Health consequences of DV**

Violence during pregnancy may affect the health of the woman and the unborn child. What do you think the risks could be of violent relationships during pregnancy?

Probes – for women, health consequences:

mental illness

social

suicide (pesticide)

physical effects

mental and psychological effects

effects on reproductive health

effects to unborn child

for men, depression

**TOPIC 5 – Men’s attitudes about human rights, laws**

How do men view women’s rights?

- What are women’s rights?
- What is the situation in Nepal for women’s education, employment, decisions about contraception/family, marital rape, trafficking, treatment of women following divorce or being widowed, etc.?
- Nepali law against domestic violence

**TOPIC 6 – Solutions to stopping violence against women**

In your opinion, what steps/actions could help eradicate violence against women?

Probes:

- changes to traditional family structures/norms, gender roles
- education and awareness
- income/jobs, women’s empowerment
- government services – legal and mental aid (free services), crisis management centres, punishment for perpetrators

**Ending –**

Is there anything that you think we have not discussed and that you would like to add? If so please feel free to do so ….

Thank you very much for your participation. Your input has been very valuable. The study results will later be disseminated to different stakeholders, including at the community level.
